# The Effect of Transcutaneous Posterior Tibial Nerve Stimulation on Pain and Quality of Life in Patients with Fibromyalgia: A Single-Blind, Randomized Controlled Trial

**DOI:** 10.3390/jcm12154989

**Published:** 2023-07-29

**Authors:** İlker Fatih Sarı, İlker İlhanlı, Tuba Mızrak, Fazıl Kulaklı, Zerrin Kasap

**Affiliations:** 1 The Department of Physical Medicine and Rehabilitation, Faculty of Medicine, Giresun University, Gazipaşa Yerleşkesi Debboy Mevki Giresun/Türkiye, Giresun 28200, Turkey; drfzl46@gmail.com (F.K.); drzerrinkasap@gmail.com (Z.K.); 2 The Department of Physical Medicine and Rehabilitation, Faculty of Medicine, Ondokuz Mayıs University, Samsun 55139, Turkey; ilkerilhanli@hotmail.com; 3 Havza Physical Therapy and Rehabilitation Center, Ondokuz Mayıs University, Samsun 55700, Turkey; tubaarslan92@gmail.com

**Keywords:** chronic pain, electric stimulation therapy, fibromyalgia, quality of life, small fiber neuropathy

## Abstract

This study aimed to investigate the effectiveness of posterior tibial nerve stimulation (PTNS) in reducing pain, improving quality of life, and decreasing disease severity in patients with fibromyalgia. This prospective, single-blind, randomized controlled trial included female patients newly diagnosed with fibromyalgia who had started duloxetine treatment (30 mg/day). The patients in the study group received six sessions of posterior tibial nerve stimulation, twice weekly, 3–4 days apart, in addition to duloxetine; the controls received duloxetine only. The patients were evaluated three times (at baseline, 1st month, and 3rd month). Pain was evaluated using a numeric rating scale, the short-form McGill Pain Questionnaire, and quality of life with a 36-item short-form health survey (SF-36). Patient functional status and disease severity were evaluated using the fibromyalgia impact questionnaire (FIQ). A total of 64 patients met the inclusion criteria: 22 were ultimately included in the study group and 30 in the control group. Statistical improvements in pain and FIQ scores were observed after treatment in both groups. The SF-36 scores indicated improved vitality only in the 1st month in both groups, with no significant changes in the other quality-of-life subscales in either group. There was no statistical difference between the two groups in terms of changes in pain, FIQ, and SF-36 scores compared with baseline at the 1st month and 3rd month. The addition of PTNS to pharmacological treatment did not contribute to the reduction in pain or improvement in quality of life in fibromyalgia either in the 1st or 3rd month. NIH Clinical Trial Registration Number NCT05937711.

## 1. Introduction

Fibromyalgia syndrome (FMS) is a musculoskeletal disease characterized by chronic widespread body pain, fatigue, sleep disturbances, and functional symptoms [1]. The etiopathogenesis of fibromyalgia has not been fully elucidated; however, central and peripheral mechanisms involving genetic and environmental factors are thought to play a role [2]. Although the most important pain mechanism is central sensitization, some evidence supports the idea that small fiber neuropathy is also involved in the pathophysiology of fibromyalgia pain [1,3]. In the peripheral mechanism of fibromyalgia, antidromic axonal reflex of C-fibers activates the peripheral neuroendocrine system. As a result of this activation, an increase in vascular permeability and vasodilation develops, and the release of neuropeptides such as glutamate and substance P increases. In addition, increased vascular permeability and bradykinin, histamine, serotonin, and local inflammatory markers released from mast cells are other mediators that cause pain in fibromyalgia [4]. In support of small fiber neuropathy resulting from this pathophysiology, higher neuropathic pain scaling values have been reported in patients with fibromyalgia, as have small fiber polyneuropathies in skin biopsies, and findings consistent with neuropathy in electrophysiological examinations [3,5,6]. In addition, drugs such as pregabalin, duloxetine, and amitriptyline, which are also used in the treatment of neuropathic pain, are also used in the treatment of fibromyalgia [7].

Peripheral nerve stimulation (PNS) is a method used in pain management and neuromodulation by targeting a nerve trunk with subcutaneous or transcutaneous electrical stimulation. Although the mechanism of action of PNS in reducing pain is still unclear, its use is increasing [8]. Neuropathic pain and other chronic pain conditions can be reduced, according to the gate-control theory, by PNS, which reduces pain by stimulating the A beta fibers and inhibiting the pain sensation carried by small fiber fibers [8,9]. When examined on the molecular level, it has been shown that when peripheral nerve stimulation is applied, neurotransmitter, endorphin, and local inflammatory mediator levels decrease. In electrophysiological studies, it has been shown that peripheral nerve stimulation reduces ectopic discharges [8,10]. Peripheral nerve stimulation has not only peripheral but also central effects. Peripheral nerve stimulation affects serotonergic, GABAergic, and glycinergic pathways [8]. PNS reduces the levels of glutamate and substance P, which are increased in the central mechanism of fibromyalgia pathogenesis [10]. Again, PNS increases serotonin and GABA levels, which decrease in fibromyalgia [10]. In imaging and animal studies, PNS affects the contralateral primary somatosensory cortex and other pain-modulating areas including the anterior cingulate, insular cortex, and thalamus [8,10]. It has been shown to inhibit the spinothalamic tract, medial lemniscal pathway, and wide dynamic range neurons in the dorsal horn suggesting that PNS modulates the supraspinal areas through the dorsal column [8,10]. 

PNS can be applied at many anatomical sites, either transcutaneously, percutaneously, or by spinal cord stimulation from electrodes implanted in the spine [11]. Transcutaneous posterior tibial nerve stimulation (PTNS) is especially used in diseases such as overactive bladder, fecal incontinence, chronic pelvic pain, and chronic prostatitis [12,13,14]. It is easy to apply, practical, and has no significant side effects. It also allows the patient to perform posterior tibial nerve stimulation on themself. In addition, PTNS is known to reduce pain in polyneuropathy [11]. Only one study in the literature has examined the effectiveness of PNS as a fibromyalgia treatment [15]. That study included only a very small number of patients, and PNS was performed via subcutaneous implantation in the C2 region [15]. 

Considering the pathophysiology of fibromyalgia and the mechanisms of peripheral nerve stimulation mentioned above, we hypothesized that posterior tibial nerve stimulation might be used in FMS. By affecting the central and peripheral mechanisms involved in the pathophysiology of fibromyalgia, which is thought to have neuropathic pain component, it was assumed that the posterior tibial nerve stimulation would reduce the pain, improve quality of life, and decrease disease severity of patients.

## 2. Materials and Methods

### 2.1. Study Design and Participants

The study was designed as a prospective, single-blind, randomized controlled (equal randomization 1:1), parallel-group clinical trial. This study was conducted at the Giresun University Giresun Training and Research Hospital and Ondokuz Mayıs University Faculty of Medicine Physical Therapy and Rehabilitation Outpatient Clinic between November 2020 and May 2022. Female patients aged between 18 and 65 years, newly diagnosed according to the American Rheumatology Association 2016 revised FMS diagnostic criteria, were included in the study. All patients started treatment with FDA-approved duloxetine (30 mg/day) according to the guidelines. Patients were excluded if they had a history of fracture/musculoskeletal surgery in the last 3 years, inflammatory joint disease, or neurological disease/neurological deficit with examination, if they were receiving medical treatment for polyneuropathy, or if they had contraindications to PNS (pacemaker, epilepsy, diminished skin sensation in the area to be applied). The study protocol and design were approved by the Clinical Research Ethics Committee of Giresun University (Decision date: 7 December 2019, number KAEK-112). All participants provided written informed consent, and the study was performed in accordance with the Declaration of Helsinki. The NIH Clinical Trial Registration Number is NCT05937711.

### 2.2. Randomization and Blinding

Patients who met the inclusion criteria and agreed to participate in the study were randomized (1:1) into two groups. Group 1 (PTNS + duloxetine) underwent six sessions of posterior tibial nerve stimulation, twice weekly, 3–4 days apart, in addition to duloxetine (30 mg/day). Group 2 (duloxetine) received duloxetine only (30 mg/day). Randomization was performed manually, with assignments placed in opaque and sequentially numbered envelopes by off-site researchers who were not involved in patient care or follow-up. Outcome measures were assessed by two investigators who were blinded to each patient’s group. The participants and nerve stimulators were not blinded to the group allocation. Patients were briefed to not disclose which group they were in during the assessment process.

### 2.3. Baseline Assessment and Outcome Measures

Sociodemographic characteristics, including age, body mass index (BMI), marital status, and education level, were recorded for the patients included in the study at baseline. The patients were evaluated three times (at baseline, 1st month, and 3rd month) by the investigator, who was blinded to each patient’s group. Pain was evaluated using the numeric rating scale (NRS) and the short-form McGill Pain Questionnaire (SF-MPQ). Pain at rest and during activity was evaluated using the NRS. The change in quality of life of the patients was calculated using a 36-item short-form health survey (SF-36). Each patient’s functional status and disease severity were evaluated using the Fibromyalgia Impact Questionnaire (FIQ).

Numeric Rating Scale: This scale was used to evaluate the patients’ general pain at rest and during activity (during activities of daily living). Patients were asked to rate their pain from 0 to 10, with 0 indicating no pain and 10 indicating the most severe pain [16].

Short-Form McGill Pain Questionnaire: This questionnaire was also used to assess the patients’ pain. This questionnaire consisted of 15 descriptive words that evaluated the sensory (11) and affective (4) dimensions of pain. Pain intensity was evaluated as follows: 0 = none, 1 = mild, 2 = moderate, and 3 = severe. Three pain scores were obtained: sensory, affective, and total (both sensory and affective). Pain felt during the survey was evaluated using a visual analog scale (VAS), and the total present pain intensity index was evaluated using a 6-point Likert rating scale (0 = no pain, 1 = mild, 2 = discomforting, 3 = distressing, 4 = horrible, and 5 = excruciating) [17].

Fibromyalgia Impact Questionnaire: This questionnaire consists of 20 questions evaluating physical function, job status, depression, anxiety, sleep, pain, stiffness, fatigue, and well-being of patients with fibromyalgia. It was scored between 0 and 100. High scores indicate high disease severity and low functional status [18].

36-item Short-Form Health Survey: This scale is commonly used to evaluate the quality of life. It consists of eight subscales (physical function, physical role limitation, pain, general health, vitality, social function, social role limitation, and mental health) and a total of 36 items. Each subscale was scored between 0 and 100, with 100 points indicating the best health condition and 0 points indicating the worst health condition [19].

### 2.4. Intervention

Patients in both groups were started on duloxetine 30 mg 1 × 1 p.o. Patients whose duloxetine treatment was discontinued or whose drug dose was increased for any reason (drug ineffectiveness, drug change, or drug intolerance) during the study were excluded.

**Figure 1:**
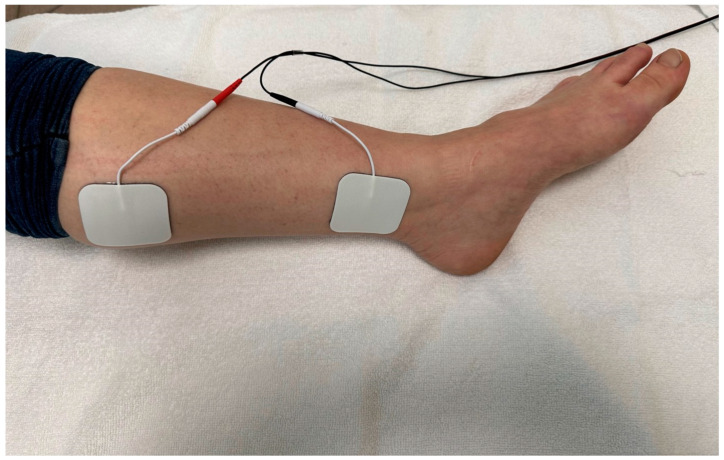
Application of posterior tibial nerve stimulation.

PTNS was applied using two 50 mm × 50 mm electrode pads per extremity. The live pad was placed superior to and medial to the medial malleolus. The ground pad was placed 5–10 cm proximal to the live pad. The application of the PTNS is illustrated in Figure 1. The PTNS was applied using biphasic square waves with a frequency of 10 Hz and pulse duration of 200 μs. The amplitude was adjusted to the level that produced painless paresthesia in each patient according to their tolerance. PTNS was applied for 30 min [20]. Patients who did not receive six sessions of treatment or who missed 3- to 4-day intervals were excluded from the study.

### 2.5. Sample Size and Statistical Analyses

The G Power (V3.1) software was used to calculate the required sample size. We could not find any previous study on PTNS in reducing pain in patients with fibromyalgia. A pilot study was conducted to determine the sample size. A pilot study was performed with 10 patients in each of the PTNS + duloxetine and duloxetine groups. Accordingly, when the changes in NRS activity values compared to baseline at 3rd month were compared between the two groups, the effect size was calculated to be 0.883. To achieve a power of 80% with a 5% probability of a type 1 error, the identified sample size was 22 patients per group based on the pilot study. Due to the COVID-19 pandemic, the loss of patients during the study period was considered to be 30%. Thus, 64 patients were included in the study.

Statistical analysis was performed using SPSS version 23.0 (IBM Corporation in Armonk, NY, USA). Continuous variables are expressed as mean ± standard deviation (SD). Categorical variables are reported as numbers and frequencies. Normality was assessed using the Shapiro–Wilk test. Quantitative data between the groups were compared using the independent samples *t*-test or Mann–Whitney U test, according to the normality of the data. Fisher’s exact test was used to compare categorical data between the groups. Intra-group comparisons were performed using one-way repeated measures analysis of variance (ANOVA) for normally distributed data or Friedman’s test as a non-parametric alternative. Post hoc analyses were performed only when significant differences were observed. In addition, changes in NRS, SF-MPQ, FIQ, and SF-36 scores from baseline at the 1st and 3rd month were compared between the groups using the independent samples *t*-test or Mann–Whitney U test. The statistical significance level was set at *p* < 0.05.

## 3. Results

**Figure 2:**
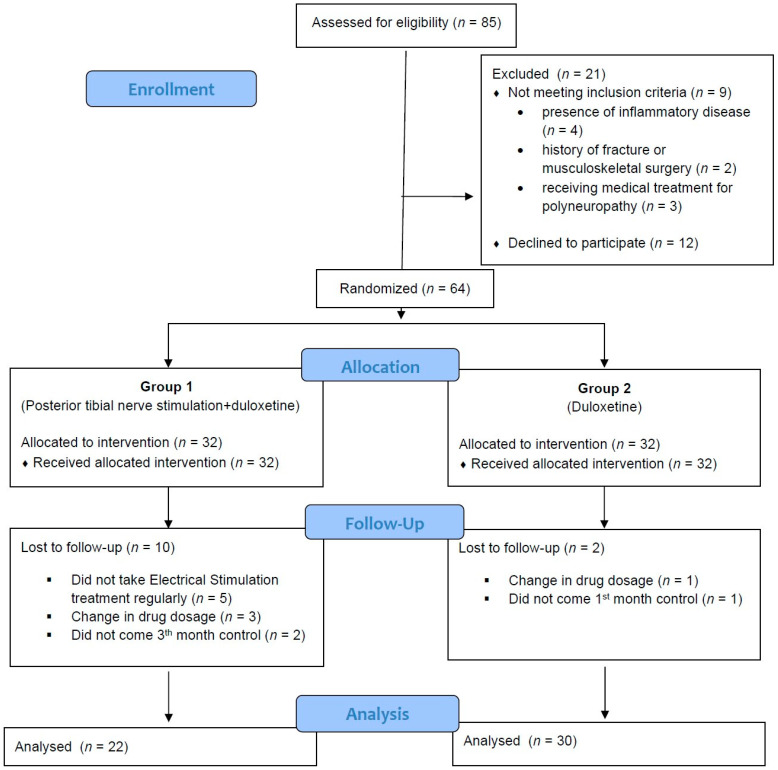
CONSORT diagram of the study.

Eighty-five patients were eligible for inclusion in this study. Of these, 12 patients did not agree to participate in the study, and 9 patients were excluded for different reasons, leaving a total of 64 patients for inclusion in the study. Eligible participants were recruited from November 2020 to May 2022. The participants attended clinic visits at the time of randomization (baseline), and at the 1st and 3rd month. The patients were randomized (1:1) into two groups. In Group 1, 5 patients who did not attend the PTNS regularly, 3 patients who had a change in the drug within 3 months, and 2 patients who did not attend the 3rd month follow-up were excluded. In Group 2, 1 patient who had a change in the drug within 3 months and 1 patient who did not attend the 1st month of follow-up were excluded, leaving 52 patients (22 in Group 1 and 30 in Group 2) for evaluation. A CONSORT diagram of the participants is shown in Figure 2. The study was ended after the 3rd month. The demographic characteristics of the study population are shown in Table 1. No differences were noted between the two groups in terms of age, sex, marital status, BMI, or smoking status.

The patients’ baseline, and 1st and 3rd month NRS rest, NRS activity, SF-MPQ, and FIQ scores are shown in Table 2. No differences were detected between the groups in terms of baseline pain scores or FIQ scores; however, a statistically significant improvement was detected in pain and FIQ scores after treatment in both groups. No statistically significant differences were detected between the two groups in terms of changes in the 1st month and 3rd month scores compared with the baseline scores (Table 2).

Evaluation of the quality of life of the patients with SF-36 showed an improvement in vitality only in the 1st month in both groups, with no significant changes evident in the other subscales of quality of life in either group. No statistical difference was noted between the two groups in terms of changes in the 1st month and 3rd months compared with the baseline scores (Table 3).

During the study, no complications occurred because of either duloxetine treatment or posterior tibial nerve stimulation. The procedure was safe, no serious adverse events occurred during or after treatment, and no patient developed infections or experienced any other events that resulted in serious harm or disability.

## 4. Discussion

The aim of this study was to investigate the effects of PTNS in FMS. The findings of this study indicate that the addition of PTNS to medical treatment did not reduce pain or fibromyalgia severity in patients with fibromyalgia.

Fibromyalgia syndrome is a disease in which chronic widespread pain is most common. The reported prevalence varies four-fold, depending on the diagnostic criteria used. It affects approximately 2–4% of the general population [21].

Although the pathophysiology of fibromyalgia remains unclear, many theories have been proposed. Central sensitization is thought to be the main mechanism leading to its pathophysiology, as it is responsible for chronic and widespread pain. However, peripheral sensitization caused by nociceptive inputs is also known to initiate central sensitization [22]. Recently, some evidence has also been presented identifying fibromyalgia as a disease of neuropathic origin that may be caused by small fiber neuropathy [3,5,6]. Some studies have reported higher neuropathic pain scale scores in patients with fibromyalgia than in controls. Skin biopsies also suggest a greater incidence of small-fiber polyneuropathy in patients with fibromyalgia than in normal controls [5,6]. Moreover, treatment drugs such as pregabalin, duloxetine, and venlafaxine, which are effective in the pharmacological treatment of fibromyalgia and are recommended as the first step, are also used to treat neuropathic pain [7]. For these reasons, fibromyalgia is suspected to be a polyneuropathy.

The efficacy of PNS in the treatment of neuropathic pain remains under investigation. PNS was introduced in the 1960s and has been used for pain control since the 1970s [11]. PNS is an effective treatment for chronic pain such as trigeminal neuralgia, episodic cluster headache, chronic migraine, complex regional pain syndrome, foot pain, and coccydynia [23]. PNS reduces pain through a gate control mechanism. PNS stimulation of large-diameter sensory fibers inhibits nociceptive impulses carried by small-diameter sensory fibers that cause pain [24]. In addition, changes in local chemical concentrations, such as the suppression of excitatory amino acids (glutamate and aspartate) and increase in inhibitory transmitters (GABA), can further reduce pain [23].

The aim of the present study was to evaluate the effectiveness of PNS in reducing fibromyalgia, a chronic widespread pain that is thought to have a neuropathic pain component for the reasons mentioned above. We found only one study in the literature in which PNS was used to treat patients with fibromyalgia disease [15]. The findings showed that an implant placed in the C2 area to control headache symptoms reduced the headache, but it also reduced widespread body pain, fatigue, and depression, and increased the quality of life at the end of the 6th month [15]. 

Transcutaneous PTNS is an easily applicable method. Also, this does not require subcutaneous placement. In addition, it was shown to be effective in urinary incontinence and was included in the treatment guidelines. Therefore, in the present study, transcutaneous PTNS was preferred; the present study is the second in which PNS has been used to treat fibromyalgia and the first in which PTNS was used. Patients who were newly diagnosed and recently started duloxetine were included in this study. As a result, significant improvement was found in both pain and FIQ scores, both in the group that received duloxetine only and in the group that received transcutaneous PTNS in combination with duloxetine. However, addition of PTNS did not significantly affect the improvement achieved with duloxetine. The reason for the previously observed effectiveness with PTNS but not effectiveness in the present study may be that patients with fibromyalgia experience pain in regions other than where PTNS is applied. Patients with polyneuropathy mostly experience pain in their distal lower extremities; therefore, applying PTNS to these regions may have helped these patients. 

Previous studies have shown that fibromyalgia negatively affects the quality of life [25,26] and that duloxetine treatment can improve the quality of life of patients with fibromyalgia [27,28]. However, we found no significant improvement in quality of life with medical treatment in our patients.

In the design of the study, one of the reasons why we preferred PTNS was that it is a self-applicable method. However, we performed it in the hospital because we thought that it would be more appropriate for the treatment to be within our control, since it was the first study in the literature. In the PTNS group, the number of dropouts was 10. The main reason why they did not continue the program was the failure to come for treatment at scheduled intervals. With further studies, we think that the dropout rate could be reduced if self-applicable transcutaneous PTNS is used. Also, we chose PTNS because it is easy to apply and has been applied many times in other diseases. However, other peripheral nerves and even multiple PNS can be tried in further studies. In our study, we performed PTNS in a certain session, with a certain duration and amplitude considering previous studies. Although the number of patients is sufficient according to the power analysis, further studies with large sample size investigating the effects of PTNS application at different sessions, durations, doses, and frequencies will contribute additional findings to the literature.

The findings of the present study are valuable because this study is the second in the literature to investigate the effect of PNS on fibromyalgia pain, and it is the first to test the effectiveness of PTNS. However, our study had some important limitations. First, only female patients were included; therefore, our findings cannot be generalized to male patients. Furthermore, medical treatment is not the only treatment option for fibromyalgia, and we did not standardize or follow any other non-pharmacological treatment options such as exercise or diet. This may also have affected treatment success. In addition, although our study was based on the notion that fibromyalgia has a neuropathic component, the fact that this component was not quantitatively evaluated using a neuropathic pain-specific test before and after the intervention is another important limitation of our study. 

In summary, PTNS was attempted for the first time as a non-pharmacological treatment for fibromyalgia, but it did not reduce the pain or overcome the negative effects of fibromyalgia by either the 1st or 3rd month of treatment. However, further studies with large sample size investigating the effects of PTNS application at different sessions, durations, doses, and frequencies, and peripheral stimulation of other nerveswill contribute additional findings to the literature.

## Figures and Tables

**Table 1:** Demographic features of the groups.

	Group 1 (Duloxetine + TPTNS) (n = 22)	Group 2 (Duloxetine) (n = 30)	*p* Value
	**Mean ± SD**	**Mean ± SD**	
Age (years)	43.18 ±12.22	49.43 ± 11.54	0.066 ^i^
BMI (kg/m^2^)	27.71 ± 4.21	28.44 ± 3.84	0.515 ^i^
	**N (%)**	**N (%)**	
Marital Status			0.639 ^f^
Married	19 (86.4)	28 (93.3)	
Single	3 (13.6)	2 (6.7)	
Education			(N/A)
Literate	3 (13.6)	0	
Elementary school	9(40.9)	16 (53.3)	
High school	5 (22.7)	11 (36.7)	
University	5 (22.7)	3 (10.0)	
Smoker			0.468 ^f^
Yes	5 (22.7)	4 (13.3)	
No	17 (77.3)	26 (86.7)	

BMI: body mass index; TPTNS: transcutaneous posterior tibial nerve stimulation; SD: standard deviation; NA: not applicable; ^i^ independent samples *t-*test; ^f^ Fisher’s exact test.

**Table 2:** Intra- and inter-group comparison of numeric rating scale, short-form McGill pain questionnaire, and fibromyalgia impact questionnaire scores.

		Group 1 (Duloxetine + TPTNS) (n = 22)	Group 2 (Duloxetine) (n = 30)	*P*_2_ Value
NRS activity	Baseline	7.68 ± 1.94	7.57 ± 2.01	0.836 ^m^
	1st month	6.05 ± 2.13 *	6.40 ± 1.89 *	0.563 ^m^
	3rd month	5.95 ± 2.01 *	6.37 ± 1.79 *	0.271 ^m^
*P*_1_ value		<0.001 ^f^	<0.001 ^f^	
NRS rest	Baseline	6.55 ± 2.18	6.40 ± 2.24	0.816 ^i^
	1st month	5.14 ± 2.08 *	5.50 ± 1.85 *	0.427 ^m^
	3rd month	5.09 ± 2.14 *	5.33 ± 1.90 *	0.718 ^m^
*P*_1_ Value		0.003 ^r^	<0.001 ^r^	
SF MPQ				
Sensory	Baseline	17.45 ± 8.50	17.63 ± 8.56	0.993 ^m^
	1st month	15.23 ± 9.11	15.03 ± 8.68 *	1.00 ^m^
	3rd month	14.05 ± 8.06 *	14.13 ± 7.74 *	0.985 ^m^
*P*_1_ Value		<0.001 ^r^	<0.001 ^f^	
Affective	Baseline	7.27 ± 3.83	7.27 ± 3.70	0.985 ^m^
	1st month	6.41 ± 3.29	6.30 ± 3.12	0.869 ^m^
	3rd month	6.05 ± 3.44 *	6.07 ± 3.28 *	0.878 ^m^
*P*_1_ Value		0.023 ^f^	0.005 ^f^	
Total	Baseline	24.73 ± 11.76	24.90 ± 11.71	0.978 ^m^
	1st month	21.64 ± 11.87	21.33 ± 11.24 *	0.985 ^m^
	3rd month	20.09 ± 10.85 *	20.20 ± 10.33 *	0.896 ^m^
*P*_1_ Value		<0.001 ^r^	<0.001 ^f^	
VAS	Baseline	7.06 ± 2.21	7.12 ± 2.22	0.941 ^m^
	1st month	5.28 ± 2.49 *	5.78 ± 2.22 *	0.676 ^m^
	3rd month	5.19 ± 2.25 *	5.69 ± 2.14 *	0.553 ^m^
*P*_1_ Value		<0.001 ^r^	<0.001 ^f^	
Present Pain Intensity	Baseline	2.64 ± 0.79	2.60 ± 0.72	0.934 ^m^
	1st month	2.00 ± 0.87 *	2.07 ± 0.83 *	0.646 ^m^
	3rd month	2.05 ± 0.72 *	2.13 ± 0.68 *	0.518 ^m^
*P*_1_ Value		<0.001 ^f^	<0.001 ^f^	0.934 ^m^
FIQ	Baseline	45.82 ± 7.81	50.20 ± 8.77	0.068 ^i^
	1st month	38.82 ± 8.57 *	46.17 ± 10.44 *	0.084 ^m^
	3rd month	34.18 ± 10.72 *	41.47 ± 13.38 *	0.167 ^m^
*P*_1_ Value		<0.001 ^r^	<0.001 ^r^	

TPTNS: transcutaneous posterior tibial nerve stimulation; NRS: numeric rating scale; VAS: visual analogue scale; SF MPQ: short-form McGill pain questionnaire; FIQ: fibromyalgia impact questionnaire; ^i^ independent samples *t*-test; ^m^ Mann–Whitney U test; ^r^ repeated measure ANOVA; ^f^ Friedman’s test; * reflects the difference with respect to baseline; *P*_1_ Value: intra-group comparison; *P*_2_ Value: inter-group comparison.

**Table 3:** Comparison of quality of life with 36-item short-form health survey.

SF-36		Group 1 (Duloxetine + TPTNS) (n = 22)	Group 2 (Duloxetine) (n = 30)	*P*_2_ Value
Physical functioning	Baseline	43.64 ± 24.11	47.00 ± 25.31	0.631 ^i^
	1st month	49.09 ± 22.18	50.00 ± 23.16	0.459 ^m^
	3rd month	46.36 ± 22.53	50.00 ± 22.89	0.960 ^m^
*P*_1_ Value		0.065 ^r^	0.217 ^r^	
Role-Physical	Baseline	22.73 ± 30.77	23.33 ± 31.44	0.951 ^m^
	1st month	17.05 ± 24.86	18.33 ± 27.02	0.973 ^m^
	3rd month	17.05 ± 28.23	19.17 ± 29.86	0.782 ^m^
*P*_1_ Value		0.325 ^f^	0.307 ^f^	
Role-Emotional	Baseline	12.12 ± 26.32	13.33 ± 28.50	0.940 ^m^
	1st month	12.12 ± 26.32	13.33 ± 28.50	0.914 ^m^
	3rd month	9.09 ± 23.42	11.11 ± 26.74	0.894 ^m^
*P*_1_ Value		0.549 ^f^	0.549 ^f^	
Vitality	Baseline	25.23 ± 18.80	25.00 ± 18.98	0.926 ^m^
	1st month	28.86 ± 17.92 *	29.00 ± 18.86 *	0.915 ^m^
	3rd month	27.95 ± 17.71	28.50 ± 19.26	0.862 ^m^
*P*_1_ Value		0.004 ^f^	0.001 ^f^	
Mental health	Baseline	43.64 ± 23.03	46.00 ± 23.62	0.720 ^i^
	1st month	49.09 ± 19.37	50.53 ± 20.49	0.858 ^m^
	3rd month	46.18 ± 21.93	48.40 ± 22.47	0.985 ^m^
*P*_1_ Value		0.128 ^r^	0.216 ^r^	
Social functioning	Baseline	41.48 ± 26.56	46.58 ± 26.40	0.495 ^i^
	1st month	49.43 ± 26.30	48.67 ± 26.09	0.482 ^m^
	3rd month	46.59 ± 28.13	48.25 ± 27.57	0.509 ^m^
*P*_1_ Value		0.294 ^r^	0.108 ^f^	
Bodily pain	Baseline	38.52 ± 18.12	39.58 ± 18.08	0.835 ^i^
	1st month	43.52 ± 24.54	44.67 ± 22.72	0.953 ^m^
	3rd month	41.59 ± 22.80	43.25 ± 21.47	0.902 ^m^
*P*_1_ Value		0.295 ^r^	0.150 ^r^	
General health perception	Baseline	32.95 ± 17.91	34.00 ± 18.63	0.925 ^m^
	1st month	31.36 ± 19.10	32.67 ± 19.77	0.906 ^i^
	3rd month	30.45 ± 18.96	32.00 ± 19.72	0.696 ^m^
*P*_1_ Value		0.254 ^r^	0.368 ^f^	

TPTNS: transcutaneous posterior tibial nerve stimulation; SF-36: 36-item short-form health survey; ^i^ independent samples *t*-test; ^m^ Mann–Whitney U test; ^r^ repeated measure ANOVA; ^f^ Friedman’s test; * reflects the difference with respect to baseline; *P*_1_ Value: intra-group comparison; *P*_2_ Value: inter-group comparison.

## Data Availability

Data can be obtained from the authors upon reasonable request.
